# Advances in essential oils for metabolic diseases

**DOI:** 10.3389/fendo.2025.1684179

**Published:** 2025-10-23

**Authors:** Xinxin Li, Min Tong, Huilian Cao, Xiangjuan Sun, Fenghua Zhang

**Affiliations:** ^1^ Clinical Medical School, Chengdu University of Traditional Chinese Medicine, Chengdu, Sichuan, China; ^2^ School of Pediatric Medicine, Henan University of Traditional Chinese Medicine, Zhengzhou, Henan, China; ^3^ Hospital of Chengdu University of Traditional Chinese Medicine, Chengdu, China; ^4^ School of Basic Medical Sciences, Chengdu University of Traditional Chinese Medicine, Chengdu, China

**Keywords:** essential oils, metabolic diseases, diabetes, obesity, mechanisms, safety

## Abstract

Metabolic diseases are a group of complex disorders caused by abnormal metabolic processes, such as diabetes, obesity, and non-alcoholic fatty liver disease. Long-term uncontrolled metabolic diseases will significantly increase the risk of cardiovascular disease, renal damage, and neurologic complications, leading to shorter survival and reduced quality of life for patients. While conventional treatments rely on pharmacological interventions, essential oils have shown significant therapeutic potential as a natural treatment modality. Essential Oils are concentrated aromatic substances extracted from plants, possessing multiple biological activities, including antioxidant, anti-inflammatory, and insulin sensitivity modulation. Studies have shown that specific essential oil components can improve metabolic disorders, enhance insulin sensitivity, and lower blood glucose, and blood lipids through a variety of mechanisms, thus playing an active role in the management of metabolic diseases. This review highlights the therapeutic potential of EOs in managing various metabolic disorders by modulating key metabolic pathways, mitigating oxidative damage, and regulating gut microbiota. We focus particularly on the rationale for selecting EOs as a research focus—their complex chemical composition enables synergistic actions against multiple pathological targets simultaneously. Additionally, we address safety profiles and current clinical evidence supporting their translational application.

## 1 Introduction

suppression and weight loss (1). The mechanism of this process involves alterations in sympathetic and parasympathetic nerve activity, as well as the regulation of leptin release (2). For example, grapefruit oil promotes lipolysis and suppresses appetite by stimulating sympathetic nerves innervating brown adipose tissue(BAT)and Metabolic diseases are a major challenge in modern medicine, representing a complex group of disorders caused by abnormal metabolic processes in the human body, involving metabolic disturbances of various biomolecules such as sugars, lipids, proteins, and minerals. The incidence of metabolic diseases is showing a significant increase and has become a global health burden (3). Epidemiological studies indicate that the global number of individuals with mellitus(DM)is projected to exceed 783 million by 2045 (4), while the obese population is expected to surpass 1 billion by 2030 (5). Concurrently, metabolism-associated fatty liver disease (6) (with a global prevalence of 38.77%) and metabolic syndrome (7) (with a prevalence of 28.2%) are also highly prevalent worldwide. Chronically unmanaged metabolic diseases will significantly increase the risk of cardiovascular complications, renal impairment, and neurological complications, leading to shorter survival and reduced quality of life. Traditional management strategies often involve lifestyle modifications and pharmacotherapy (8), yet these may be limited by side effects, cost, and accessibility.

In recent years, Essential Oils (EOs) have gained scientific attention as complementary natural products with potential metabolic benefits. Derived from various plant parts through steam or hydro-distillation, EOs are complex mixtures of volatile compounds such as terpenes, phenolics, and aromatic molecules (9, 10). Their bioactive constituents enable multi-mechanistic actions—including anti-inflammatory, antioxidant, and metabolic enzyme modulation—making them particularly suitable for addressing the multifactorial nature of metabolic diseases (11–13). Unlike single-target pharmaceuticals, EOs possess a broad spectrum of activity that simultaneously influences interconnected pathways such as insulin signaling, lipid metabolism, and oxidative stress response.

This article aims to review the current research advances in the use of EOs for the treatment of metabolic diseases and to evaluate the associated safety considerations and available clinical data.

## 2 Essential oils

**Figure 1:**
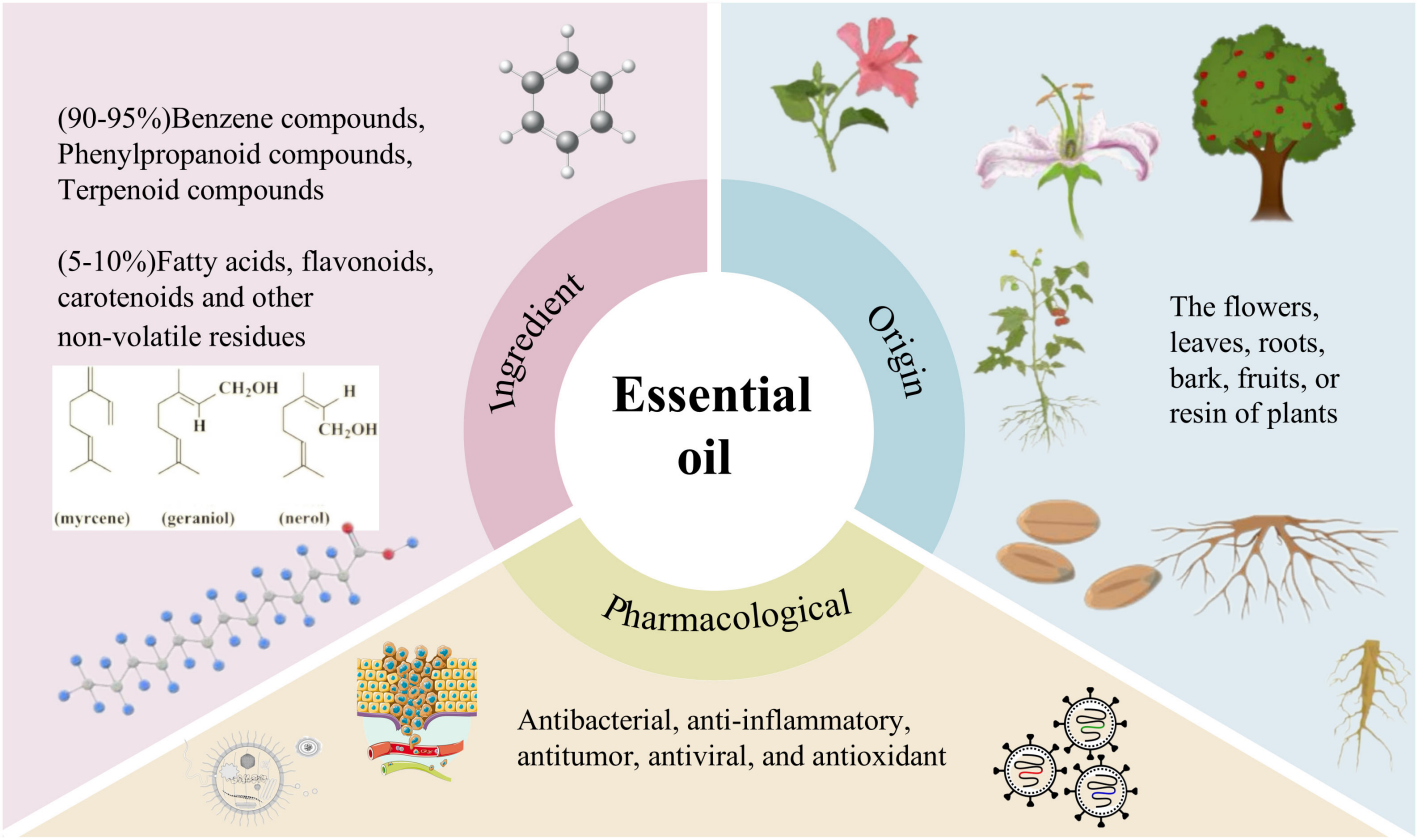
Components, sources, and pharmacology of essential oil.

EOs are highly concentrated, naturally occurring aromatic compounds extracted from the flowers, leaves, roots, bark, fruits, or resins of plants, usually obtained by steam distillation or hydro distillation (**Figure 1**). EOs are colorless, volatile liquids that are soluble in organic solvents, most of which have strong odors, and consist of a complex natural mixture of 20–60 ingredients (9). In pure EOs, the volatile fraction accounts for about 90-95% of the total weight (**Figure 1**), with the main compounds being benzenes, phenylpropanoids, and terpenoids; the other 5-10% are nonvolatile residues containing fatty acids, flavonoids, carotenoids, and hydrocarbons (10). Terpenes are the largest class of chemicals found in EOs and are made from 5-carbon isoprene units. Among terpenes, monoterpenes and sesquiterpenes are the most abundant in EOs (14). Monoterpenes can become linear or cyclic compounds through redox reactions, and monoterpenes can generate other compounds with typical functional groups such as alcohols, aldehydes, ketones, esters, and ethers (15). Sesquiterpenes can also exist as hydrocarbons or contain oxygen functional groups, including carboxylic acids, lactones, alcohols, aldehydes, ketones, and epoxides. EOs have been widely studied for their therapeutic potential in various pathologies, and their pharmacological profile includes antimicrobial (**Figure 1**), anti-inflammatory, antitumor, antiviral, and antioxidant activities (14). EOs are capable of influencing the metabolic processes of the body through a variety of bioactive actions. EOs can enhance insulin sensitivity and ameliorate metabolic disorders (16). EOs exert their antioxidant effects by modulating antioxidant enzymes and reducing lipid peroxidation, thereby alleviating oxidative stress (OS) and providing adjunctive therapeutic effects in DM (17). Studies have shown that EOs can regulate lipid metabolism, inhibit adipocyte differentiation, and reduce fat accumulation (18–20). The anti-inflammatory effects of EOs can also reduce chronic inflammation (CI), and treat obesity and related metabolic disorders by targeting adipose tissue inflammation (21). In addition, studies have shown that EOs may indirectly regulate metabolic processes by influencing the composition and function of the gut microbiota, thereby effectively preventing the progression of metabolic diseases such as fatty liver and DM (22–24).

## 3 Pathogenesis of metabolic diseases

The pathogenesis of metabolic diseases is multifactorial and multilayered, usually involving the interaction of IR, CI, abnormalities in lipid metabolism, and gut microorganisms imbalance.

Insulin resistance (IR) is a central link in metabolic diseases, referring to reduced sensitivity of target tissues to insulin (25), which impairs glucose uptake and utilization and leads to compensatory hyperinsulinemia. IR not only promotes the development of type 2 diabetes mellitus (T2DM) (26) but also contributes to non-alcoholic fatty liver disease (NAFLD) (27) by enhancing hepatic lipid synthesis and inhibiting fatty acid oxidation, while exacerbating obesity (28) through the promotion of lipogenesis and suppression of lipolysis. Chronic inflammation (CI), often originating from adipose tissue in obesity, releases excessive free fatty acids, reactive oxygen species, and pro-inflammatory cytokines (29), which interfere with insulin signaling, worsen IR (30), increase the risk of plaque rupture and thrombosis (31), and drive the progression of NAFLD from simple steatosis to non-alcoholic steatohepatitis (NASH) (32). Oxidative stress (OS), characterized by excessive reactive oxygen species (ROS) production under conditions of hyperglycemia, lipotoxicity, and inflammation, further impairs pancreatic β-cell function and insulin signaling pathways, promotes lipid peroxidation and vascular endothelial dysfunction (33, 34), thereby forming a vicious cycle of OS-IR-CI. Dyslipidemia—marked by elevated triglycerides(TG), low-density lipoprotein(LDL), cholesterol, and reduced high-density lipoprotein (HDL)—further drives cardiovascular complications and hepatic steatosis (35, 36). Finally, gut microbiota dysbiosis indirectly influences IR, lipid metabolism, and systemic inflammatory status by modulating the production of metabolites such as short-chain fatty acids and bile acids, thereby regulating host energy metabolism, inflammatory responses, and intestinal barrier function (37, 38).

These pathways are interconnected, forming a self-perpetuating vicious cycle of metabolic dysfunction. The multi-target properties of EOs make them promising agents capable of disrupting these cycles at multiple points simultaneously.

## 4 Research progress and application of EOs in different metabolic diseases

### 4.1 Diabetes mellitus

**Figure 2:**
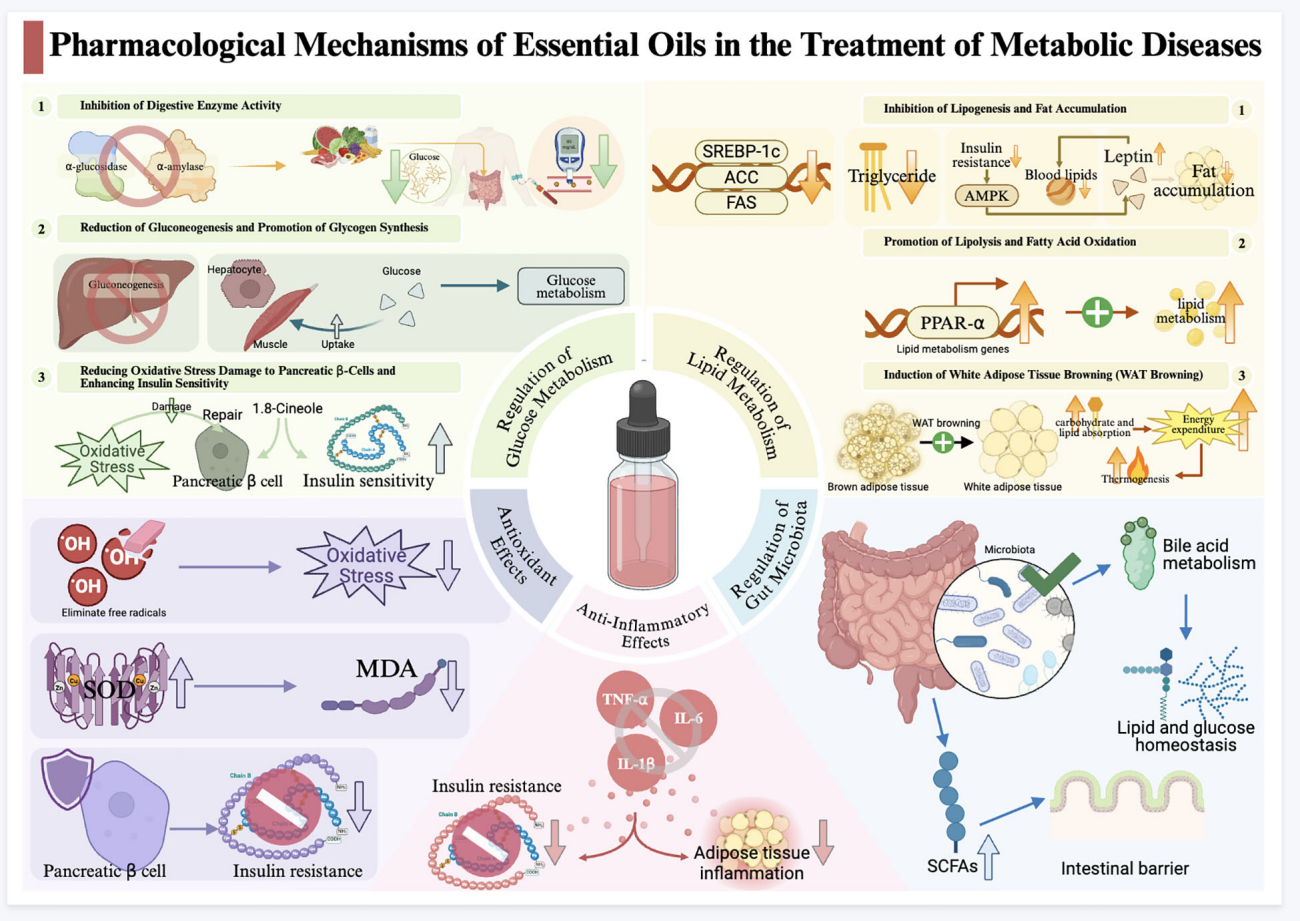
Pharmacological mechanisms of essential oils in the treatment of metabolic diseases. SOD, superoxide dismutase; MDA, malondialdehyde; TNF-α, tumor necrosis factor-α; IL-6, interleukin-6; IL-1β, interleukin-1β; SREBP-1C, sterol regulatory element-binding protein-1c; ACC, acetyl-coa carboxylase; FAS, fatty acid synthase; AMPK, AMP-activated protein kinase; PPAR-α, peroxisome proliferator-activated receptor α; SCFAs, short-chain fatty acids. Up arrows indicate increased activity and down arrows indicate decreased activity of the process.

Many bioactive phytoconstituents, such as carvacrol, thymol, and α-pinene, have been shown to have positive effects on DM management (39). Inhibition of digestive enzyme activity, reduction of gluconeogenesis, and enhancement of sensitivity to insulin or insulin secretion are considered to be the main mechanisms underlying the anti-DM activity of EOs (17)(**Figure 2**). Specifically, by inhibiting α-glucosidase and α-amylase activities, the absorption of sugar from the digestive tract can be effectively reduced, thus preventing the appearance of postprandial hyperglycaemic peaks. Studies have shown that the inhibitory effect of EO of galangal flower (**Table 1**) on α-glucosidase was comparable to that of a positive control drug, acarbose (40), whereas Lavandula officinalis EO exhibited significant inhibitory activity on both digestive enzymes (41). In rat experiments, the application of fennel EO (**Table 1**), both orally and topically, significantly reduced blood glucose levels in diabetic model rats, which was associated with its intervention on enzymes involved in glucose metabolism (e.g., α-amylase and α-glucosidase), which in turn affects the secretory function of the pancreas and regulates intracellular glucose uptake (42).

**Table 1:** EO for metabolic diseases (the table presents the component of the EOs reviewed, their main mechanisms of action targeting relevant metabolic diseases, and associated references).

Disease types	EO	Component of EO	Targets and details	References
DM	Galangal flower EO	Farnesene, farnesyl acetate, aceteugenol	Inhibits α-glucosidase activity	(40)
	Lavandula officinalis EO	L-camphor, 1,8-cineole, borneol	Anti-inflammatory, hypoglycemic, inhibits α-glucosidase and α-amylase activity.	(41)
	Fennel EO	Trans-anethole, fenchone	Lowering blood glucose, inhibiting α-glucosidase and α-amylase activity	(42)
	Citrus aurantifolia Leaf EO	D-limonene, neral, linalool, sulcatone, isogeraniol	Reduces gluconeogenesis and promotes gluconeogenesis	(45)
	Fructus Alpiniae Zerumbet EO	1,8-Cineole	Antioxidant, mitigates mitochondrial dysfunction and apoptosis, restores β-cell function.	(47)
	Lemongrass EO	α-citral, Geraniol, Geranyl acetate, neral	Antioxidant, Hypoglycemic	(48)
	Nigella sativa oil	Monoterpenes	Antioxidant, anti-inflammatory, maintains pancreatic β-cell integrity	(49)
Obesity	Grapefruit EO	Limonene	Stimulates sympathetic nerves and inhibits parasympathetic gastric nerves, promoting lipolysis and suppressing appetite	(50)
	Peppermint EO	(L)-menthol, (L)-menthone	Suppress appetite	(2)
	Thyme EO	Thymol Durenol	Suppress appetite	(2)
	Litchi flower EO	Alcohols, FA, esters, alkanes, alkenes	Promotes nematode movement and increases energy expenditure	(51)
	Lemongrass EO	α-citral, Geraniol, Geranyl acetate, neral	Lower triglyceride levels	(52)
	Basil EO	Linalool oxide, linalool, 1-menthene, carvone	Reduces leptin resistance	(53)
	Garlic EO	Allicin	Increases leptin levels and reduces fat deposition	(54)
	Cinnamon EO	Cinnamaldehyde	Stimulates the pancreas to release insulin, which inhibits adipocyte differentiation and adipogenesis	(19)
	Ginger EO	[6]-gingerol [8],-gingerol [10],-gingerol [6],-shogaol	Activates AMPK signaling pathway, increases leptin and HDL-cholesterol levels, and enhances skeletal muscle lipolysis	(55)
	Fennel EO	Trans-anethole	Increased beige-specific gene expression induces browning of 3T3-L1 adipocytes, increases mitochondrial biogenesis in WAT, and activates BAT.	(57)
		Farnesol	Induction of thermogenic factor expression and promotion of lipolytic enzymes	(58)
NAFLD	Citrus peel EO	α-limonene, α-pinene, α-myrcene, α-phellandrene	Down-regulation of SREBP-1c and ACC, up-regulation of LXRα and CYP7A1, and reduction of hepatic TC and TG levels in rats	(59)
	Turmeric EO	Curcuminoids	Enhanced expression of lipid metabolism gene PPAR-α inhibits hepatic fat accumulation.	(60)
	*Active ingredients from Eriobotrya japonica EO*	Nerolidol	Reduces steatosis, hepatocyte degeneration, and infiltration of inflammatory cells, and reduces hepatic lipid accumulation	(61)
	*Amomum villosum EO*		Improvement of liver histology and lipid accumulation, anti-inflammatory, antioxidant	(62)
	Pink lotus EO	Palmitic acid ethyl ester, linoleic acid ethyl ester	Regulation of lipid metabolism, anti-inflammation, enhancement of insulin sensitivity, and alleviation of FFA-induced steatosis in HepG2 cells	(63)
AS	Lemon EO	Limonene, β-pinene, γ-terpinene	Reduces TC, promotes LDL oxidation and lipid peroxidation, prevents erythrocyte deformation and aortic endothelial proliferation	(65)
	Rosmarinus officinalis L. EO	1,8-cineole, camphor, limonene, α-pinene	Reduced TC, LDL, and TG levels and inhibited vascular endothelial AS progression	(66)
	Fructus Alpinia zerumbet EO	α-pipene, β-pinene, camphor, α-cadinol	Binds to PPARγ protein, activates PPARγ-LXRα-ABCA1/G1 pathway, promotes cholesterol efflux, improves lipid metabolism, anti-inflammation	(67)
	Active compounds from zedoaria rhizoma	Zedoarondiol	Regulation of the CXCL12/CXCR4 pathway inhibits monocyte migration and adhesion to endothelial cells.	(68)
	A monoterpene naturally found in EOs	Eucalyptol	Anti-inflammatory, upregulates LDL receptor (ABCA-1) expression, promotes cholesterol transport from macrophages, and attenuates AS	(69, 70)
	Ginger EO	Citral	Anti-inflammatory, inhibits AS	(71)
		Citronellal	Anti-oxidative stress, anti-inflammatory, and reduction of carotid atherosclerotic plaque size in AS rats	(72)
	Citri Reticulate Pericarpium EO	Flavonoids	Antioxidant, prevents AS formation	(73)
Thyroid Dysfunction	Nigella sativa EO	P-cymene, thymoquinone, α-thujene, longifolene, β-pinene, α-pinene, carvacrol	Bidirectional regulation of thyroid-stimulating hormone, triiodothyronine, and thyroxine levels, and antioxidants	(75–77)
	Olive oil	Triacylglycerols, secoiridoid glycosides, phenolics, flavonoids	Thyroid stimulating activity	(78)
	Petroselinum crispum EO	*α*-pinene, myristicin, apiole	Inhibits oxidative stress and maintains thyroid function	(79)
	EOs		Relief of thyroid-related symptoms such as fatigue, anxiety, and depression	(80)

The complete **Table 1** is formatted in the same three-line style throughout.

A new cellular study has found that novel preparations containing phenolic compounds (one of the active ingredients in EOs) can regulate glucose metabolism by significantly reducing hepatic gluconeogenesis and increasing glucose uptake by muscle and liver cells (43). Several *in vivo* experiments provide strong evidence for this mechanism. For example, Cissus quadrangularis extract showed hypoglycaemic effects in a leptin receptor mutant db/db mouse model by a mechanism that involves inhibition of the expression of proteins associated with gluconeogenesis (e.g., insulin receptor substrate 1 and AMP-activated protein kinase) and glucose-6-phosphatase, and facilitation of glycogen synthesis, which results in lower hepatic glucose levels (44). The potential of EOs in regulating hepatic glucose metabolism was further demonstrated by Fatima et al. By intervening with Citrus aurantifolia Leaf EO (**Table 1**) in hyperglycaemic rats, it was found that intraperitoneal administration (at a dose of 100 mg/Kg) for a sustained period of 14 days significantly lowered fasting blood glucose and hepatic glucose levels, as well as significantly increased hepatic glycogen concentration. This suggests that the main mechanism of action of EOs lies in reducing gluconeogenesis and and facilitation of glycogen synthesis (45). Complementary studies have also found that certain EO constituents such as thymoquinone can effectively inhibit the gluconeogenesis process by precisely targeting specific metabolic enzymes (46).

In addition to regulating glucose metabolism, EOs can reduce damage to pancreatic β-cells from OS, enhance insulin sensitivity(**Figure 2**), and slow the progression of diabetic complications. A study on Fructus Alpiniae Zerumbet EO (**Table 1**) showed its restorative effect on β-cell function. The main active ingredient of this effect, 1,8-Cineole, directly binds to and enhances the stability of Sirtuin1, thus correcting the impaired oxidative homeostasis, inhibiting mitochondrial dysfunction and apoptosis, which are common in T2DM, and ultimately restoring β-cell function (47). Lemongrass EO (**Table 1**) improves blood glucose levels in diabetic rats by effectively regulating the redox state through its powerful free radical scavenging ability (48), while Nigella sativa oil (**Table 1**) excels in inhibiting OS and inflammatory responses in T2DM, helping to maintain pancreatic β-cells integrity and improve insulin sensitivity (49).

### 4.2 Obesity

In recent years, there has been a gradual increase in research on EOs in obesity management, focusing on metabolic regulation and appetite control. Whether inhaled or oral, EO therapy has shown anti-obesity potential. It has been found that specific compounds in EOs regulate the expression of appetite-related neuropeptides in the hypothalamus by interacting with the olfactory system, as evidenced by a decrease in appetitive hormone production and a decrease in the mRNA expression of appetite-producing neuropeptides, as well as an increase in appetite-suppressing neuropeptides, leading to appetite adrenal glands, and inhibiting parasympathetic gastric nerves. Its main component, limonene, exerts this effect through a histaminergic response (50). In addition, EOs such as peppermint, thyme (**Table 1**), and citrus aurantium have all been shown to have appetite-suppressant effects (2).

EOs also exert anti-obesity effects by regulating metabolism. By intervening with litchi flower EO (LFEO) (**Table 1**) in nematodes, Chen Yun and other researchers found that LFEO treatments of 10, 20, and 30 μg/mL significantly reduced TG content and promoted nematode locomotion to increase energy expenditure, thus regulating lipid metabolism (51). A cellular model experiment showed that EOs such as lemongrass oil, ginger oil, and black pepper oil significantly inhibited lipid and TG accumulation in the range of 12-24%, while also decreasing lipid and TG levels in mature adipocytes. By analyzing their active ingredients, limonene was found to have the strongest inhibitory effect on lipid accumulation (**Table 1**), while citral and camphene significantly reduced TG levels in adipocytes (52). Linalool (another major constituent in EO) has similarly shown to inhibit lipid accumulation by down-regulating the mRNA expression of sterol regulatory element-binding protein 1, as well as its target gene fatty acid synthase and acetyl-coenzyme A carboxylase, and up-regulating the mRNA expression of genes associated with fatty acid oxidation (18).

Adipokines such as leptin and insulin play an important role in the regulation of appetite and energy metabolism, and their resistance usually leads to the development of obesity. A variety of EOs show potential to modulate these factors. For example, inhalation of basil EO (**Table 1**) reduced leptin levels, food intake, and body weight in rats, which is thought to be a result of reduced leptin resistance (53). In addition, the use of garlic EO (**Table 1**) similarly increased leptin levels and decreased fat deposition (54). Cinnamon EO (**Table 1**) is rich in cinnamaldehyde, which stimulates the release of insulin from the pancreas and inhibits adipocyte differentiation and adipogenesis, thus exerting an anti-obesity effect (19). Ginger oil (**Table 1**) has been shown to reduce IR, activate the AMPK signaling pathway, increase leptin levels, and reduce blood lipids, which has a positive impact on the prevention and treatment of obesity (55).

Under certain conditions, WAT can be converted to BAT, a phenomenon known as ‘WAT browning’. It has been shown that activating BAT and inducing WAT browning accelerates glycolipid uptake and reduces insulin secretion requirements (56), essentially increasing energy expenditure by activating thermogenesis(**Figure 2**). The unique chemistry of EOs offers the potential to facilitate this process. Trans-anethole (TA), the main component of fennel EO, has shown promising anti-obesity properties. Kang et al. showed that TA treatment of C57BL/6 mice increased the expression of beige-specific genes such as Cd137, Cited1, Tbx1, and Trem26. TA induced browning of 3T3-L1 adipocytes by activating β3-AR as well as the AMPK-mediated SIRT1 pathway that regulates PPARα and PGC-1α, increased mitochondrial biogenesis in WAT, and activated BAT to exhibit thermogenic activity. Also, TA regulates lipid metabolism in white adipocytes by inhibiting lipogenesis and liposynthesis and increasing lipolysis and oxidation (57). Similarly, Farnesol (**Table 1**), a naturally occurring 15-carbon organic compound widely found in various EOs, has been shown to have similar effects (58), further enriching the research horizon of EOs in metabolic regulation.

### 4.3 Non-alcoholic fatty liver disease

EOs can regulate lipid metabolism in the liver due to their active ingredients. An *in vivo* experiment showed that oral administration of citrus peel EO (**Table 1**) (CPEO) at concentrations of 0.5% and 0.75% significantly reduced hepatic total cholesterol (TC) and TG levels, and ameliorated hepatic steatosis and accumulation of lipid droplets in rats fed a high-fat diet. Lipidomic analysis showed that this alteration was associated with the downregulation of genes related to lipogenesis (e.g., SREBP-1c, ACC, and FAS) and the upregulation of genes related to bile acid biosynthesis (e.g., LXRα, CYP7A1, and CYP27A1) in the liver (59). In addition, the hexane fraction of turmeric EO (**Table 1**) has been shown to enhance the expression of the lipid metabolism gene PPAR-α (**Figure 2**), thereby promoting lipid metabolism and inhibiting fat accumulation in the liver (60). Further studies have shown that Sabir et al. observed the effect of feeding a nerolidol diet on a rat model of NAFLD and showed that nerolidol treatment significantly reduced steatosis, hepatocyte degeneration, and infiltration of inflammatory cells, which reduced lipid accumulation in the liver and prevented the progression of the disease due to a high-calorie diet, thus inhibiting further development of nonalcoholic steatohepatitis (61).

The protective effect of EOs on the liver is also reflected in their unique anti-inflammatory and antioxidant mechanisms. A 56-day intervention with 2 g/kg of Amomum EO(EOA) in tilapia showed that this treatment improved liver histology and lipid accumulation, enhanced antioxidant capacity, and reduced inflammation. Further analyses indicated that EO elevated peroxisomal enzyme activity and reduced the level of inflammatory indicators, which decreased oxidative damage and inflammatory responses in the liver by attenuating OS and oxidative damage to cell membranes (62). In addition, Pink lotus EO (PLEO) from lotus flowers reduced TC and TG levels in FFA-treated HepG2 cells. The analysis showed that PLEO inhibited the excretion of tumor necrosis factor-α (TNF-α), interleukin-1β (IL-1β), and interleukin-6 (IL-6), decreased the phosphorylation of lipid metabolism enzymes (**Figure 2**), and increased the phosphorylation of phosphatidylinositol3-kinase (PI3K) and NF-κB. The study revealed for the first time that PLEO (**Table 1**) exerts a hepatoprotective effect by regulating lipid metabolism, inhibiting inflammatory responses, and increasing insulin sensitivity to alleviate FFA-induced steatosis in HepG2 cells (63).

### 4.4 Atherosclerosis

Metabolic disorders drive the pathogenesis and progression of AS (64), making it a key cardiovascular complication of metabolic diseases. Evidence from several studies suggests that plant-derived EOs may be useful in lowering cholesterol levels and regulating lipid metabolism. For example, lemon EO effectively reduces TC levels in hypercholesterolemic rabbits and prevents erythrocyte deformation and aortic intima proliferation by promoting LDL oxidation and lipid peroxidation (**Figure 2**). The major oil components limonene, β-pinene, and laurin greatly influence lipid metabolism, thereby ameliorating the damage caused by hypercholesterolemia (65). Similarly, Rosmarinus officinalis EO (**Table 1**) and its nanoemulsion exhibited positive effects on dyslipidaemic rats, significantly reducing TC, LDL, and TG levels and inhibiting vascular endothelial AS progression (66). In a mouse model of AS, the EO from Fructus Alpinia zerumbet (EOFAZ) was observed to improve lipid metabolism and attenuate inflammatory markers. Its chemical components directly bind to the peroxisome proliferator-activated receptor γ (PPARγ) protein, which in turn activates the PPARγ-LXRα-ABCA1/G1 pathway to inhibit foam cell formation, thereby exerting anti-AS effects (67).

EOs inhibit monocyte migration, reduce inflammatory mediators, and alleviate vascular endothelial inflammatory responses. Zedoarondiol, an active compound extracted from zedoaria rhizoma (**Table 1**), was found to ameliorate AS plaques in the facial aorta and its roots in mice. Its mechanism of action is to effectively inhibit the migration and adhesion of monocytes to endothelial cells by modulating the CXC chemokine ligand 12/CXC chemokine receptor 4 pathway (68). Eucalyptol or 1,8-Cineole is a monoterpene found in a variety of plant EOs with a wide range of biological activities. In a study on diabetic AS rats, Eucalyptol treatment significantly reduced the levels of inflammatory mediators (tumor necrosis factor α) and prevented the formation of AS (69). Eucalyptol, a hepatic X receptor agonist, upregulates LDL receptor expression, facilitates cholesterol transport from macrophages, and reduces the levels of inflammatory mediators (70). In addition, ginger oil (**Table 1**) has been shown to inhibit inflammatory cytokines in plasma, particularly interleukin-1β, and therefore inhibit the formation of AS to some extent (71).

Citronellal, a monoterpene derived mainly from plant secondary metabolism, was effective in reducing the size of carotid atherosclerotic plaques in AS rats. It inhibits OS and inflammation in the vascular endothelium and improves endothelial dysfunction. Its antioxidant effects are mediated by modulating the activity levels of nitric oxide, malondialdehyde, and superoxide dismutase (72). Flavonoids from Citri Reticulate Pericarpium (**Table 1**) have also demonstrated potent antioxidant capacity by scavenging free radicals, hydrogen peroxide, and chelating ferrous ions, thereby preventing AS formation (73).

### 4.5 Thyroid dysfunction

Thyroid hormones are central regulators of metabolic health, modulating energy metabolism—including lipid synthesis and breakdown, cholesterol metabolism, glucose metabolism, and thermogenesis—across multiple tissues such as the liver, adipose tissue, muscle, and heart (74). Dysregulation of thyroid hormone signaling pathways directly contributes to the pathogenesis and progression of various metabolic diseases. Consequently, thyroid dysfunction serves as a key driver and significant comorbidity in metabolic disorders. EOs have shown great potential as a natural complementary therapy in regulating thyroid function. Nigella sativa EO (**Table 1**) (NSE) extracted from Nigella sativa Linn seeds is rich in a variety of active compounds, such as thymoquinone, α-thujene, and carvacrol, which not only possess antifungal, antibacterial, and antioxidant properties (75) but also show unique advantages in thyroid function regulation. Through experimental studies, the researchers used a 200 mg/kg dose of NSE to intervene in the rat model of thyroid dysfunction induced by propylthiouracil and L-thyroxine to observe its modulatory effects. The experimental results revealed the bidirectional regulatory mechanism of NSE: in the HT rat model, thyroid-stimulating hormone (TSH) level decreased significantly, and the concentrations of triiodothyronine (T3) and thyroxine (T4) increased markedly; whereas, in the HP rat model, the opposite trend of change was observed. More importantly, NSE also effectively improved the OS state in the model and alleviated the oxidative damage induced by thyroid dysfunction (76). Further studies have shown that NSE is effective in restoring thyroid hormone levels and endocrine signaling under the influence of sodium fluoride toxicity, which makes it particularly important in protecting and re-establishing thyroid function (77). In addition to NSE, Olive oil (78) and Petroselinum crispum EO (**Table 1**) (79) have likewise been shown to have positive effects on thyroid function regulation. Recent clinical studies further support the therapeutic value of EOs in alleviating thyroid-related symptoms such as fatigue (80), anxiety, and depression.

Although extensive preclinical studies, including cellular and animal models, have revealed the potential mechanisms of EOs in regulating glucose and lipid metabolism, improving IR, and alleviating OS, clinical evidence remains insufficient. Human trials focusing on metabolic diseases are limited in number, and existing clinical research predominantly emphasizes the adjunctive alleviation of comorbid emotional disorders—such as anxiety, depression, and sleep disturbances (81)—rather than core pathophysiological metabolic indicators. Moreover, most current studies incorporate EOs as part of multimodal interventions, making it difficult to isolate their independent therapeutic effects. Therefore, this article does not provide a detailed separate discussion on the clinical efficacy of EOs in metabolic diseases to avoid misleading interpretations resulting from inadequate evidence strength.

## 5 Safety, toxicological assessment of EOs

EOs are highly concentrated volatile aromatic compounds extracted from plants, characterized by their high concentration, lipophilicity, and complex chemical composition. Most EOs are safe when applied appropriately and in correct doses (81). However, exceeding safe dosage limits may lead to adverse reactions or toxic effects.

EOs contain various sensitizing chemical constituents (e.g., linalool, limonene). Direct application of undiluted products may cause skin irritation, burns, or allergic reactions (82). Furocoumarins present in citrus oils such as bergamot and lemon exhibit phototoxicity (83). Dermal exposure to ultraviolet radiation after application may result in severe sunburn, blistering, or hyperpigmentation. Certain EOs containing monoterpenes such as thujone, camphor, and α-terpinene have demonstrated neurotoxicity in *in vitro* and *in vivo* studies (84). High-dose ingestion may affect the central nervous system, leading to convulsions and other severe symptoms. Prolonged or excessive use of some EOs may also cause hepatotoxicity. Furthermore, due to the potential maternal toxicity, teratogenicity, and embryotoxicity associated with many EOs (85), their use should be avoided during pregnancy and lactation. The use of EOs must strictly adhere to the following safety guidelines to minimize potential risks: EOs should always be diluted with a carrier oil before use, and direct application of undiluted oils is prohibited. Oral ingestion should be avoided unless under professional guidance. Special populations, including pregnant and lactating women, infants, young children, and individuals with chronic diseases, must consult healthcare professionals before use. Sun exposure should be avoided for at least 12 hours after applying citrus oils to prevent phototoxic reactions. Additionally, high-quality, pure EOs should be selected and stored in a cool, dark place, out of reach of children.

In summary, the safety of EOs depends on the method of application, dosage, and individual conditions. Although potential risks exist, these can be effectively managed through scientific toxicological evaluation and adherence to safety principles such as proper dilution and dose control, allowing for the safe utilization of their benefits.

## 6 Conclusion

This review highlights the significant potential of EOs as multi-target agents in the management of metabolic diseases. Their complex chemical composition enables synergistic effects on insulin sensitivity, lipid metabolism, inflammation, and OS—all of which represent key pathways implicated in DM, obesity, NAFLD, AS, and thyroid disorders.

However, current research continues to exhibit significant limitations. Although preclinical studies have provided promising mechanistic insights into the application of EOs in metabolic diseases, a clear disconnect exists within the evidence base: a wealth of *in vitro* and animal data starkly contrasts with the limited number and inconsistent quality of clinical studies. The current number of clinical trials is insufficient, and there is a lack of systematic research to further validate the actual effects of EOs in humans. Most studies feature small sample sizes and a scarcity of large-scale, long-term randomized controlled trials. Furthermore, inherent methodological limitations in preclinical research, such as inadequate adherence to principles like randomization and blinding, coupled with the chemical complexity of EOs and batch-to-batch variations, contribute to uncertainties and challenges in reproducing results. The heterogeneity in research methodologies and the significance of inter-individual metabolic differences further limit the generalizability of the findings. Concurrently, inconsistent observations in the literature—for instance, divergent reports on the modulation of key hormones like leptin by different EOs—highlight the potential influences of species differences, dose-response relationships, and chemical compositional variability. The field notably lacks head-to-head comparative studies designed to directly evaluate the efficacy of different EOs. More critical knowledge gaps remain, including the lack of sufficient validation regarding optimal dosage, routes and frequency of administration, and an unclear understanding of the synergistic effects between different EO constituents. The absence of human pharmacokinetic data, unclear long-term safety profiles, and unevaluated risks of interactions with conventional medications also hinder clinical translation. Due to the high volatility and bioactivity of EOs, further research is needed to ensure the quality and stability of EOs and related formulations. Currently, safety analyses focusing on EOs for treating metabolic diseases are relatively scarce. Therefore, toxicological studies should be strengthened to systematically assess their impact on human health through precise qualitative and quantitative analyses.

Future research directions should focus on the following aspects: First, increasing the number of clinical trials and *in vivo* experiments to bridge the translational gap between preclinical and clinical research. Second, expanding sample sizes, standardizing research methodologies, systematically evaluating dosage and administration routes, and conducting comprehensive toxicological studies and randomized controlled trials to deeply explore the molecular mechanisms of EOs in regulating metabolism in conditions like obesity. Third, utilizing modern omics technologies and multi-center clinical studies to construct precise risk-benefit assessment models, develop standardized EO formulations for interventions, and establish personalized treatment strategies. The ultimate goal is to form a robust scientific evidence system based on evidence-based medicine to comprehensively advance the safe and effective application of EOs in the management of metabolic diseases.
